# Differential Toxicity of Bare and Hybrid ZnO Nanoparticles in Green Pea (*Pisum sativum* L.): A Life Cycle Study

**DOI:** 10.3389/fpls.2015.01242

**Published:** 2016-01-12

**Authors:** Arnab Mukherjee, Youping Sun, Erving Morelius, Carlos Tamez, Susmita Bandyopadhyay, Genhua Niu, Jason C. White, Jose R. Peralta-Videa, Jorge L. Gardea-Torresdey

**Affiliations:** ^1^Environmental Science and Engineering, The University of Texas at El PasoEl Paso, TX, USA; ^2^University of California Center for Environmental Implications of Nanotechnology, The University of Texas at El PasoEl Paso, TX, USA; ^3^Texas A&M AgriLife Research Center at El PasoEl Paso, TX, USA; ^4^Department of Analytical Chemistry, The Connecticut Agricultural Experiment StationNew Haven, CT, USA; ^5^Department of Chemistry, The University of Texas at El PasoEl Paso, TX, USA

**Keywords:** bare, doped, coated, ZnO nanoparticles, phytotoxicity, dissolution, seed quality

## Abstract

The effect of surface or lattice modification of nanoparticles (NPs) on terrestrial plants is poorly understood. We investigated the impact of different zinc oxide (ZnO) NPs on green pea (*Pisum sativum* L.), one of the highest consumed legumes globally. Pea plants were grown for 65 d in soil amended with commercially available bare ZnO NPs (10 nm), 2 wt% alumina doped (Al_2_O_3_@ZnO NPs, 15 nm), or 1 wt% aminopropyltriethoxysilane coated NPs (KH550@ZnO NP, 20 nm) at 250 and 1000 mg NP/kg soil inside a greenhouse. Bulk (ZnO) and ionic Zn (zinc chloride) were included as controls. Plant fresh and dry biomass, changes in leaf pigment concentrations, elements (Zn, Al, Si), and protein and carbohydrate profile of green pees were quantified upon harvest at 65 days. With the exception of the coated 1000 mg/kg NP treatment, fresh and dry weight were unaffected by Zn exposure. Although, all treated plants showed higher tissue Zn than controls, those exposed to Al_2_O_3_@ZnO NPs at 1000 mg/kg had greater Zn concentration in roots and seeds, compared to bulk Zn and the other NP treatments, keeping Al and Si uptake largely unaffected. Higher Zn accumulation in green pea seeds were resulted in coated ZnO at 250 mg/kg treatments. In leaves, Al_2_O_3_@ZnO NP at 250 mg/kg significantly increased Chl-*a* and carotenoid concentrations relative to the bulk, ionic, and the other NP treatments. The protein and carbohydrate profiles remained largely unaltered across all treatments with the exception of Al_2_O_3_@ZnO NPs at 1000 mg/kg where sucrose concentration of green peas increased significantly, which is likely a biomarker of stress. Importantly, these findings demonstrate that lattice and surface modification can significantly alter the fate and phytotoxic effects of ZnO NPs in food crops and seed nutritional quality. To the authors' knowledge, this is the first report of a life cycle study on comparative toxicity of bare, coated, and doped ZnO NPs on a soil-grown food crop.

## Introduction

Engineered nanoparticles (ENPs), due to their high surface to volume ratio and greater numbers of atoms at the particle surface, have been widely used in the fields of medicine, agriculture (nano-fertilizers and nano-pesticides), manufacturing, electronics, and energy production (Ghormade et al., 2011; Roco, 2011; Bandyopadhyay et al., 2013; Gardea-Torresdey et al., 2014). It has been estimated that the global nanotechnology market will exceed to $3 trillion by the year 2020 (Venkatesan et al., 2004). In recent years, hybrid ENPs, e.g., doped and coated nanomaterials (NMs), have received increased attention due to their potential applications in microelectronics, semiconductors, optical device fabrication, and optics (Venkatesan et al., 2004; Ozgur et al., 2005; Dhiman et al., 2012). Commercially, available silane coupling agent (KH550) coated ZnO NPs and alumina doped (Al_2_O_3_) ZnO NPs are two of the important hybrid NPs and are being used in the fabrication of detectors and optoelectronic devices (Zhang et al., 2010; Thandavan et al., 2015), preparation of novel polymer- inorganic nanocomposites, among others (Abdolmaleki et al., 2012). Unique properties, such as, high reactivity and bio-compatibility are two reasons for concern related to potential toxicity to biota. The rapidly increasing production and use have elevated the likelihood of ENP exposure in the environment (Mukherjee et al., 2014a,b). However, very little is known about the environmental health and safety of these newer hybridized materials.

The literature has shown mixed effects of NP exposure on various animals, plants, and microorganisms; depending upon their species, growth conditions, NP type and exposure concentrations, among others. For example, Montalvo et al. (2015) reported improved phosphorus bioavailability through the application of hydroxyapatite nanoparticles to wheat (*Triticum aestivum).* Application of nanomaterials toward nano-fertilizer development and plant disease suppression is described elsewhere (Liu and Lal, 2015; Servin et al., 2015). Conversely, ample evident of negative effects could also be found in the literature. For example, growth can be negatively affected by ENPs exposure (Lin and Xing, 2007; Sinha et al., 2011; Bandyopadhyay et al., 2012a,b; Gaiser et al., 2012; Hawthorne et al., 2012; Mukherjee et al., 2014b; Rico et al., 2014). There are several reports on the toxicity of different ENPs on food crops (Lin and Xing, 2007; Lee et al., 2008; Navarro et al., 2008; Sinha et al., 2011; Bandyopadhyay et al., 2012a,b; Gaiser et al., 2012; Hawthorne et al., 2012; Zhao et al., 2013a, 2014a; Rico et al., 2014; Mukherjee et al., 2014a,b). However, a mechanistic understanding of the impact of ENPs on edible/crop plants is needed for accurate exposure and risk assessment, but this knowledge remains elusive. “The Nanotechnology Consumers Products Inventory” identifies zinc oxide (ZnO) NP as the fifth most widely used material in terms of use in the consumer products (Maynard and Evan, 2006). ZnO NPs are commonly used in personal care products, anti-microbial agents, paints, and photovoltaics (Szabo et al., 2003; Hernandez-Viezcas et al., 2013). However, ZnO NPs have been shown to be potentially toxic in the environment (Kahru and Dubourguier, 2010). For instance, a 5-day exposure study with ZnO NP-DI water suspension in petri dishes showed root growth inhibition in ryegrass (*Lolium perenne*), radish (*Raphanus sativus*), and rape (*Brassica napus*) (Lin and Xing, 2007). NPs can also exert phytotoxicity by disrupting the water and nutrient pathways in plants (Szabo et al., 2003; Lin and Xing, 2008; Kahru and Dubourguier, 2010; Lopez-Moreno et al., 2010; De La Rosa et al., 2011). Lopez-Moreno et al. (2010) reported on the genotoxicity of ZnO NPs to soybean (*Glycine max*). A reduction in wheat (*Triticum aestivum*) biomass upon ZnO exposure, along with elevated reactive oxygen species (ROS) level, was reported by Dimkpa et al. (2012). Zhao et al. (2013a) observed reduction in chlorophyll production in corn (*Zea mays*) grown in soil amended with ZnO NPs at 800 mg/kg. Importantly, the toxicity of ZnO NPs may often be due to its greater dissolution or release of Zn^2+^ ions into the growth media as a function of small particle size, opposed to the induction of oxidative stress by the parent ENPs (Hendry and Jones, 1980; Nel et al., 2006; Xia et al., 2006; Du et al., 2011; Kim et al., 2011; Priester et al., 2012). For example, released Zn^2+^ ions from the dissolution of ZnO NPs can displace the central Mg^2+^of chlorophyll, effectively disabling the photosynthetic core, causing phytotoxicity (Rebeiz and Castelfranco, 1973; Hendry and Jones, 1980; Kupper et al., 1996; Oberdorster et al., 2005). There are very few reports on the effects of NPs on seed quantity, quality, or nutritional content. For instance, CeO_2_ NPs change the nutritional quality of wheat (*Triticum aestivum* L.) (Rico et al., 2014). The fruit quality of soybean was impacted by ZnO and CeO_2_ NPs (Priester et al., 2012). However, there appears to be no information available on the comparative toxicity of bare, doped, and coated ZnO NPs on green pea (*Pisum sativum* L.).

The aim of this work was to evaluate the effect of surface coating and lattice doping on ZnO NP-plant interactions. Green pea was chosen because of its high global consumption and nutritional value (Iqbal et al., 2006). Pea plants were exposed to different concentrations of ENPs and bulk ZnO and zinc chloride. The accumulation/uptake of Zn, Al (present in doped NP), and Si (present in KH550 coating) in different plant tissues, as well as the mineral, carbohydrate, and protein content in seeds were also determined.

## Materials and methods

### Soil sampling

The soil was collected from the field at Texas AgriLife Research Center, El Paso, TX (31°41′44.98″N; 106°17′ 01.36″ W, top 20 cm) and is a sandy loam with 3.73% clay, 12.15% silt, and 84.1% sand (Zhao et al., 2013a). The experiment was conducted in a 1:1 mixture of the native soil with high organic matter potting soil [Miracle-Gro Garden Soil for Flowers & Vegetables; N-P-K = 0.09-0.05-0.07] so as to improve the soil quality in terms of soil porosity, and water retention capacity, among others.

### Pot preparation

The bare ZnO NPs (10 nm commercial spheroid, Meliorum Technologies, New York) were obtained from the University of California Center for Environmental Implications of Nanotechnology (UC CEIN). Two percent wt Al_2_O_3_@ZnO (15 nm), and one percent wt KH550 coated ZnO NPs (20 nm) were obtained from US Research Nanomaterials (http://www.us-nano.com). ENPs and bulk ZnO were added as dry powder at 0 (control), 250 and 1000 mg NPs/kg of soil in black plastic containers (Ns-400; diameter: 20 cm; tall: 12.5 cm; volume: 3.925 L; Nursery Supplies). To achieve 1 wt% dissolved Zn, equivalent amount of 5 and 20 mg/kg zinc chloride was dissolved in 50 mL Millipore water (MPW) and added to the soil for ionic treatments. The soil was vigorously mixed with spatulas to maximize particle/ion homogeneity. Early rise variety of green pea (Seeds of Change, USDA organic, Home Depot, life cycle 65 days) were immersed in 4% bleach solution and rinsed three times with tap water. Seeds were soaked overnight in regular tap water and were sown in the test pots for a 65-day growth period. Two hundred milliliters of nutrient solution per day [0.72 g/L 15 N− 2.2 P− 12.5 K (Peters 15-5-15); EC = 1.80 dS/m; pH = 6.62] was added to each pot and the pots were maintained for 24 h in the green house for stabilization. The daily light integral (photosynthetically active radiation) was 15.3 ± 3.1 mol/m^2^/d. The greenhouse temperature was maintained at 26.9 ± 8.6°C during the day and 13.7 ± 4.3°C at night. The relative humidity was 41.6 ± 19.1%.

### Zeta potential, size, and pH of the NP suspensions

Particles were dispersed in 10 mL Millipore water (MPW) to achieve 250 and 1000 mg/L concentrations, sonicated for 10 min, and kept undisturbed for 1 h and the zeta potential and size were measured using a Zetasizer Nano-ZS 90, (Malvern Instruments Ltd., UK). The pH of the supernatants was measured. Each analysis was performed in triplicate.

### Dissolution of different NPs in soil solution

Release of Zn^2+^ was measured by dispersing all the NPs and bulk ZnO in soil solution containing 5 g of soil mixture (1:1) in 20 mL DI water at a concentration of 1000 mg/kg soil. Zinc chloride was excluded due to its complete solubility in water. Each measurement was done in three replicates at three sampling intervals of 15, 30, and 45 days. These samples were used for the time-dependent dissolution study. Multiple serial centrifugations were used to remove suspended particles from the solution and to isolate the dissolved Zn ions. At the predetermined time (15, 30, and 45 days) intervals, samples were taken and centrifuged at 5000 rpm (Eppendorf AG bench centrifuge 5417R, Hamburg, Germany), and 2 mL aliquot of the supernatant was collected and centrifuged at 14000 rpm for 30 min. Subsequently, this supernatant was transferred and centrifuged again at 14000 rpm for 45 min. This process was repeated three times to remove particulate matters (Bandyopadhyay et al., 2015). The final supernatant was diluted to 15 mL with 2% HNO_3_ and elemental concentrations were measured using ICP-OES/MS as described below.

### Elemental analysis of soil, plant tissues, and seeds

For each replicate, 1 g of native and 1:1 soil were collected separately from the stock pile and grounded in mortar-pestle. Approximately, 200 mg of soil portions were digested in a microwave acceleration reaction system (CEM MARSx, Mathews, NC) with 1:4 plasma pure HNO3 (trace metals ≤ 1 ppb) and H_2_O_2_ at 195°C for 30 min (ramp 5 min; hold 25 min) in triplicate (Packer et al., 2007). Sixty five-day old pea plants were harvested and roots were washed with 0.01 M HNO3, with subsequent rinsing in DI water. The tissues were then oven dried at 70°C for 2 days (Fisher Scientific Isotemp., Pittsburgh, PA; USA). The seeds were dried at room temperature for a week. Different tissues were weighed and digested similar to that described above. The digested samples were analyzed for elemental content using a Perkin Elmer optima 4300 DV inductively coupled plasma optical emission spectrometer (ICP-OES) or ICP-MS (ELAN DRC II; Perkin-Elmer) as required.

### Chlorophyll and carotenoid estimation in leaf

Approximately 0.5 gram fresh, razor blade chopped leaves were placed into 15 mL tubes. Five mL acetone was added and the samples were shaken overnight on a horizontal shaker (Revco Scientific DS1473AVA, 115 volts, 60 Hz, 7 amps). The supernatants were collected and absorbance was measured at 470, 645, and 662 nm using a Perkin Elmer Lambda 14 UV/Vis spectrometer (single-beam mode, Perkin-Elmer, Uberlinger, Germany). Concentrations of Chl-*a, b*, and total carotenoids were measured according to a previously described method (Wellburn, 1994).

### Determination of starch, total soluble sugars, and reducing sugars in seeds

The total soluble sugar extraction was performed following the method of Verma and Dubey (2001) with little modification. A sample of 100 mg of dried pea seed was ground in 2 mL of 80% ethanol and then heated (80°C) in a water bath for 30 min. After cooling to room temperature, the extracts were centrifuged at 14000 rpm for 30 min (Thermo Scientific, Soruall T1, U.S.); a process that was repeated twice. All supernatants were combined and the total soluble sugar content was determined spectrophotometrically (λ = 490 nm, single-beam mode, Perkin-Elmer, Uberlinger, Germany) following the method of Dubois et al. (1956). The reducing sugar content was measured spectrophotometrically (λ = 620 nm) by the procedure of Somogyi (1952). In both cases, sugar content was determined against a standard calibration curve of glucose. The amount of non-reducing sugar was determined by subtracting the value of reducing sugar from total sugar.

Seed starch was also determined following the method of Verma and Dubey; the residue from total sugar extraction was used to measure the starch content (Verma and Dubey, 2001). The precipitate was dried at 70°C for 24 h, 2 mL of MPW was added, and the mixture was heated in a water bath at 95°C for 15 min. After cooling to ambient temperature, 1 mL of concentrated sulfuric acid was added. The suspension was stirred for 15 min, and the final volume was adjusted to 5 mL using MPW. The supernatant was centrifuged at 3000 rpm for 20 min, and the extraction was repeated once using 50% sulfuric acid. The supernatants were combined and diluted to 10 mL. The starch content was quantified following the method of Dubois et al. (1956) and expressed in mg/100 g dry weight.

### Protein fractionation in seeds

Protein fractionation was performed according to Chen and Bushuk (1970). Dried pea seeds (100 mg) were extracted sequentially with 2 mL each of water, 0.5 mol/L NaCl, 70% ethanol, and 0.05 M acetic acid for 2 h. The extracted protein in each step was labeled as albumin (water soluble), globulin (salt-soluble), prolamin (alcohol-soluble), and glutelin (acid-soluble), respectively. Each fraction was centrifuged at 14,000 rpm; the supernatants were collected and analyzed using the methods of Bradford (1976).

### Statistical analysis

Unless otherwise noted, all the treatments were replicated four times. Data (means ± SE) were reported as averages of four replicates. We have run the Two-way ANOVA considering treatment type and concentration as variables with 6 and 2 levels respectively, followed by Tukey-HSD multiple comparison for the means to check the individual effects and their interactions at *p* ≤ 0.05 (R version 3.1.3). Pairwise comparison tests (with adjusted *p*-values) between concentration and nanoparticle type revealed high significance in some cases. However, we plotted only those interactions which resonate with the research goals, promote clarity and ease of comparison.

## Results and discussion

### Size, zeta potential, and pH of different particles in MPW

In MPW, doped NPs had lower hydrodynamic diameter values than the other particles (Table S1). As expected, at 250 and 1000 mg/L, bulk ZnO possessed significantly higher diameter (1627 ± 198.9 and 9324 ± 236.8 nm) followed by coated (526.6 ± 14.2 and 608.5 ± 11.9 nm), bare-ZnO (397.5 ± 25.3 and 290.9 ± 20.2 nm), and doped NPs (362.2 ± 20.7 and 244.1 ± 25.6 nm). Interestingly, as the concentration increased, the size of aggregates of bare and doped nanoparticles decreased but that of the coated NP increased. This may be due to higher rate of aggregation and co-precipitation of bare and doped NPs with other suspended NPs and/or soil particles at the higher concentration, leaving behind smaller aggregates in the suspension. Conversely, the coated NP, due to its high negative surface charge (−26.4±7.09 mV, Table S1), can form hydrogen bonding in MPW, leading to greater stability of the dispersion with larger NPs in diameter compared to bare and doped forms.

With the exception of coated nanoparticles, all particles showed positive zeta potential. The order of magnitude was: coated < bare-ZnO < doped < bulk. The higher zeta potential for doped NPs compared to bare-ZnO NPs can be attributed to the fact that in doped NPs, Al^3+^ replaced Zn^2+^ in the ZnO lattice, which increases the surface potential. The negative zeta potential of the coated nanoparticles is understandable, given the nature of the surface coating; The ethoxy groups present in aminopropyltriethoxy-silane (KH550) can hydrolyze readily in water and generate hydroxyl silane (Wang et al., 2012). Thus, the attachment of KH550 onto the surface of ZnO NPs and corresponding hydrolysis could create a negative surface charge through the activity of the oxygen atoms, yielding a negative zeta potential. Although, there are differences in the numerical values of soil pH (7.7–8.5) among the treatments, these differences are not statistically significant (Table S1). This might be due to fewer number of replicates (three) and/or short exposure period.

### Particle dissolution

**Figure 1:**
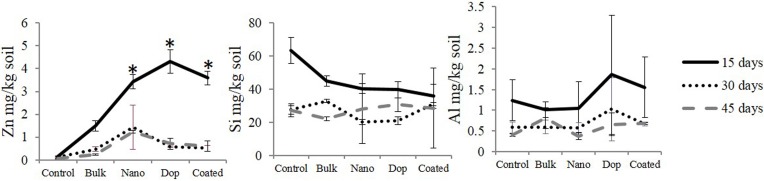
**Zinc, silicon, and aluminum dissolution from all the particles after 15, 30, and 45 days at 1000 mg/kg soil concentration**. Data points with same/no symbols (^*^) represent no statistical significance at *p* ≤ 0.05.

Dissolution data (mg/kg soil) of all the treatment is shown in Figure 1. We found no significant differences in dissolution across the three types of NPs in soil suspension (Figure 1). Similarly, the amount of released Si and Al remained unaffected at a given time, most probably, due to: (i) the very low amount of Al and Si in doped (2 wt%) and coated (1 wt%) NPs, respectively, with regard to the mass of ZnO NP and (ii) high background concentrations of Si and Al coming from the soil could make it difficult to quantify the source-specificity (NP vs. soil) of those two elements. On the other side, a variation in Zn dissolution was observed with time. For instance, at 15th day, the amount of released Zn for all three NPs varied from 3.4 to 4.3 mg/kg soil but this difference was not enough to reach statistical significance. As expected, bulk ZnO particles released 1.5 mg Zn/kg soil, which was significantly less than all nano treatments (*p* ≤ 0.05). This could be attributed to the larger size of the bulk particles, which yields far less surface area and subsequently, less dissolution from the ZnO. The amount of dissolved zinc did not change between 30 and 45 days. Interestingly, the extent of dissolution after 30 and 45 days was lower than that of 15 days. This may be due to the production of zinc hydroxide that precipitates from solution and/or sorption of zinc ions to the different soil components, leaving behind fewer zinc ions upon reaching equilibrium (Wang et al., 2013; Zhao et al., 2013a). It is important to note that the dissolution study was neither intended to guide the toxicological experiments nor to elucidate the dissolution kinetics, but to check the trend of NP-dissolution in this particular soil type. It is noteworthy that we assumed 1 wt% zinc dissolution, although our dissolution study showed 0.06–0.43 wt% dissolution. The reasons for this difference are that the current study was performed under a closed system whereas the actual experiment was conducted under constant irrigation; this could significantly increase zinc dissolution. This is in part why we preferred to consider a “higher” amount reported in the literature, which was 1 wt% of ZnO NP (Bian et al., 2011). To achieve ~1 wt% of zinc ion, we added 2 wt% of ZnCl_2_ as the molar mass of Zn (65.4) is close to half the molar mass of ZnCl_2_ (136.3). As such, 5 and 20 mg ZnCl_2_/kg soil approximate 1 wt% Zn^2+^ dissolution from 250 and 1000 mg ZnO NPs /kg soil, respectively.

### Fresh/dry weights and zinc/aluminum/silicon bioaccumulation in root/stem/leaf

The elemental analysis of the native soil and 1:1 soil mix is shown in Table S2. Significant changes were observed in the elemental composition between the native and 1:1 soil (Table S2). The total amount of Zn, K, Mg, S, Mn, P, and Mo increased with the amendment of organic matter rich potting soil. However, the Fe concentration decreased and the Ca and Cu concentrations were unaltered. There was a slight numerical decrease in the pH values in the native (7.7 ± 0.18) and 1:1 soil (7.2 ± 0.08) but the difference was not of statistical significance.

The biomass of plants exposed to Zn treatments is shown in Figure S1. At 250 mg/kg treatments, regardless of particle type, the fresh and dry weight were unaffected by Zn treatment. Similarly, at 1000 mg/kg, nano, doped and ion-exposed plants had equivalent biomass, compared to the unexposed controls. However, the 1000 mg/kg bulk and coated NP treatments significantly increased the fresh weight, relative to the control plants; although the same trend was seen for dry weight, the differences were not statistically significant (Figure S1).

**Figure 2:**
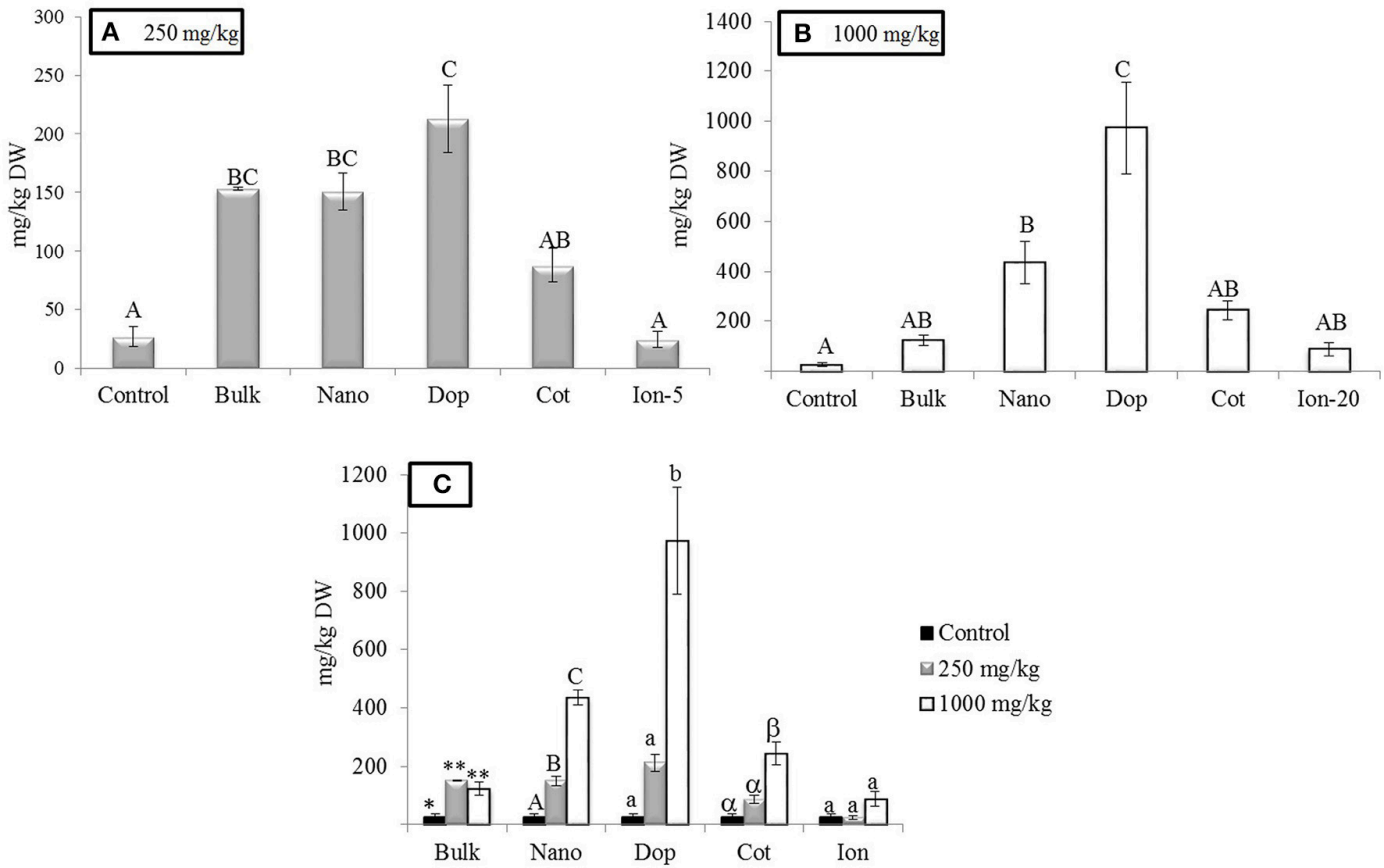
**Zinc bioaccumulation in root tissues**. **(A,B)** Show the effects of different treatments at 250 and 1000 mg/kg exposure, respectively. **(C)** Shows the comparison among control, 250, and 1000 mg/kg treatments for each type of treatment separately. Bars are mean ± SE. Bars with same letters/symbols represent no statistical significance at *p* ≤ 0.05. Upper case, lower case, and symbols are mutually exclusive.

As expected, Zn treatments increased root Zn (Figure 2). At 250 mg/kg exposure, roots showed 5.8, 5.8, and 3 times more Zn for bulk, bare, and coated NPs, respectively, compared to controls. The doped NP exposure yielded a root Zn concentration 8 times higher than controls. Moreover, increases at 1000 mg/kg bare NP and doped treatments were 16–36 times higher than controls (Figure 2). The level of Zn in the 5 mg/kg ion exposed (“Ion-5”) plants was equivalent to that of the control. The bulk, coated, and ion exposures did have nominal concentrations that were higher than the controls but large variability among these specific replicates resulted in statistical insignificance in these treatments. Concentration dependent increases in Zn content were evident for the nano, doped, and coated treatments; these trends were less clear for the bulk and ion exposures.

**Figure 3:**
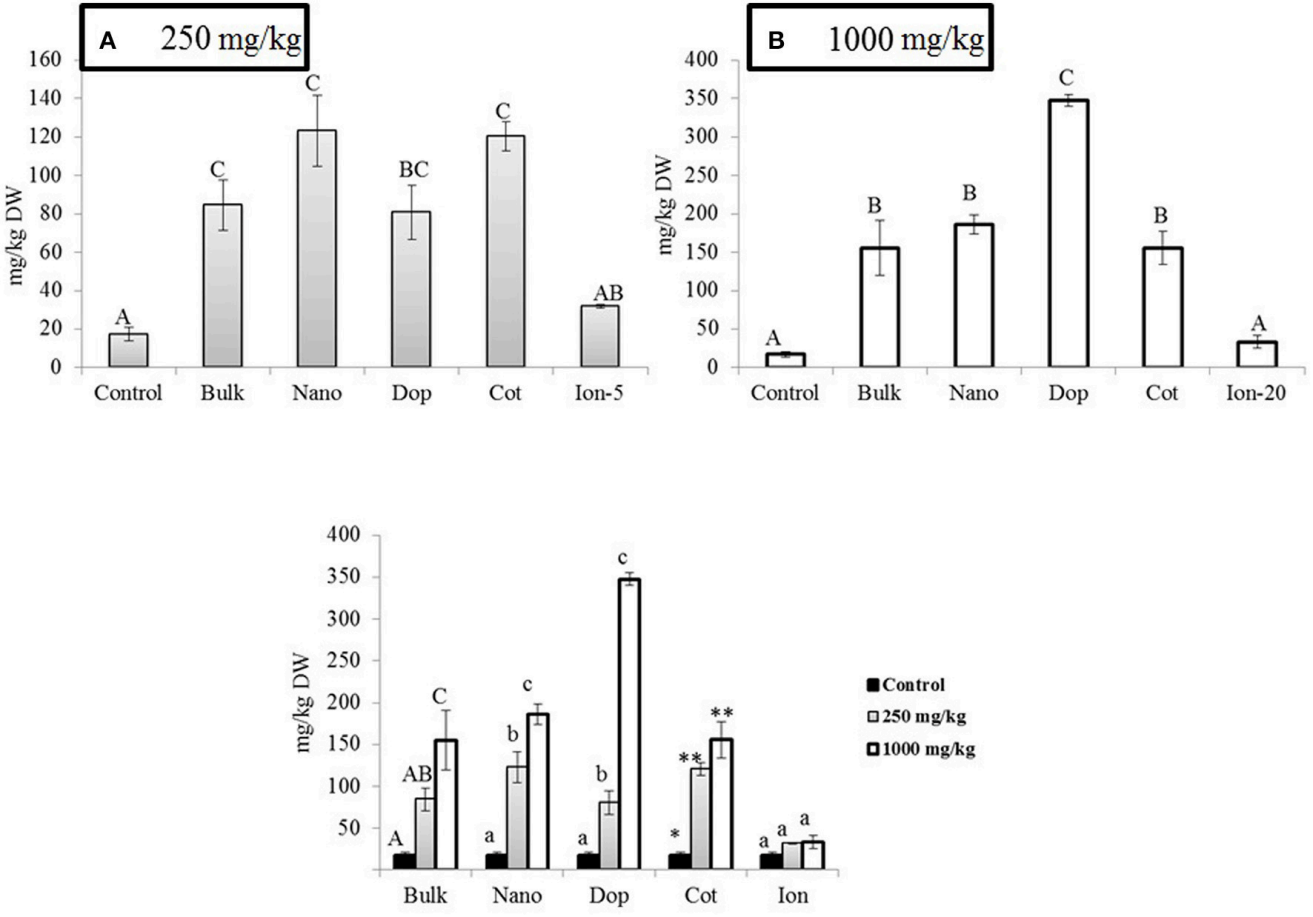
**Zinc bioaccumulation in stem tissues**. **(A,B)** show the effects of different NP treatments at 250 and 1000 mg/kg exposure, respectively. The bottom graph shows the comparison among control, 250, and 1000 mg/kg treatments for each type of treatment separately. Bars are mean ± SE. Bars with same letters/symbols represent no statistical significance at *p* ≤ 0.05. Upper case, lower case, and symbols are mutually exclusive.

**Figure 4:**
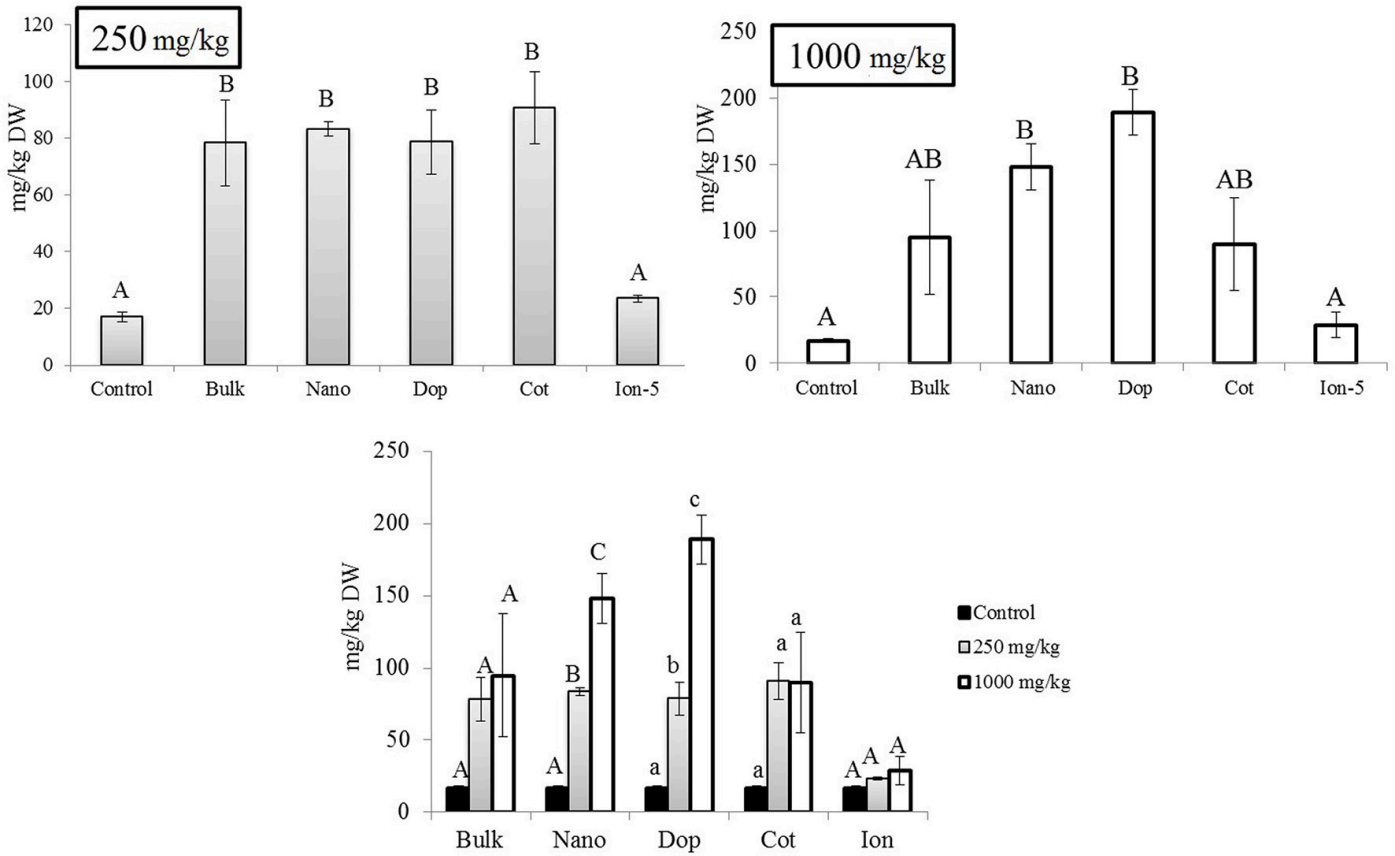
**Zinc bioaccumulation in leaf tissues**. Top graphs show the effects of different NP treatments at 250 and 1000 mg/kg exposure, respectively. The bottom graph shows the comparison among control, 250, and 1000 mg/kg treatments for each type of treatment separately. Bars are mean ± SE. Bars with same letters represent no statistical significance at *p* ≤ 0.05. Upper case, lower case, and symbols are mutually exclusive.

Similar to the roots, green pea stems showed significant increase in Zn accumulation upon exposure, with the exception of the ionic zinc treatment (Figure 3). Increased Zn accumulation was in the following order: at 250 mg/kg, with the increases relative to control stems expressed parenthetically: bulk (5x), bare (7x), doped (4.7x), and coated (7x); at 1000 mg/kg, the values were as follows: bulk (9x), bare (11x), doped (20x), and coated (9x) (Figure 3). Unlike the roots, at 250 mg/kg there were no significance differences across the nanoparticle treatments. However, at 1000 mg/kg, similar to the roots, the accumulation of Zn from the doped nanoparticle treatment was significantly greater than the other nanoparticles. Similar to the roots, all ZnO treatments exhibited concentration dependent increases in zinc at the two exposure levels; this trend was not evident for the ion exposure. In leaves, all amendments except the ion treatment showed 4.6–5.3 fold increases in zinc uptake with exposure at 250 mg/kg but there were no differences among the particle types. At 1000 mg/kg, only the nano and doped treatments resulted in values significantly above the controls (5.5–11 times; Figure 4).

No concentration dependent changes in Al and Si uptake were observed. Al and Si uptake by pea roots, stems, and leaves were largely unaffected across the different treatments. However, a few exceptions to this overall trend were noted. In stems, at the 1000 mg/kg treatments, doped and coated NPs accumulation showed 2.7 to 3.3 fold decreases in Al uptake compared to control (Figure S2). Similarly, silicon uptake into pea roots was decreased significantly at 250 mg/kg bare (2.6x) and doped (2x) treatments compared to control (Figure S3). In roots at 1000 mg/kg, bare nanoparticle exposure resulted in a 2.4 times decrease in Si content but no other differences were of statistical significance compared to control.

Previously, Mukherjee et al. reported the differential effects of bare-ZnO NPs, bulk ZnO, and iron doped ZnO (Fe@ZnO) NPs on green peas cultivated in a growth chamber (Mukherjee et al., 2014a,b). At 250 mg/kg, in all the tissues, bulk and bare-ZnO NPs showed similar 3–6 fold increases in zinc uptake compared to control. However, in agreement with our current data, at higher concentrations (500 mg/kg) bare-ZnO NP showed 2.5 to 4 times higher Zn bioaccumulation compared to bulk treatment (Mukherjee et al., 2014a). Conversely, Mukherjee et al. (2014b) reported that roots of green pea exposed to 500 mg/kg Fe@ZnO showed lower Zn uptake (9x) compared to the NP treatment (12x). In addition, our current findings also indicate an opposite trend with a 36 fold increase in Zn uptake at higher concentrations of alumina doped, compared to bare-ZnO NPs. Therefore, changes in the doping agents (i.e., alumina or iron) can clearly change the uptake behavior of Zn in higher plants. Increases in element uptake from Al_2_O_3_@ZnO treatment compared to Fe@ZnO could be attributed to i) higher (more positive) surface charge due to alumina doping, which ensures greater adhesion/absorption to the root surface and ii) higher ion dissolution. At 1000 mg/kg, ZnO NPs@KH550 showed less (5x–9x) uptake across all tissues compared to all other particles. This might be attributed to larger size in soil and high negative surface change which exerts a repulsive force to the negatively charged root surface. Additionally, silicon has been proven to reduce the bioavailability of zinc ions in plants (Gu et al., 2011). Therefore, silicon released from the dissolution of coated NPs (KH550 or 3-aminopropyltriethoxysilane) may be another cause for reduced Zn uptake compared to bare and doped NPs. In case of bare ZnO NP, intermediate size, and zeta potential could be two of the most important governing factors for keeping the extent of zinc uptake in-between doped and coated NPs. Although we do not know the reason for different Zn accumulation as a function of particle/exposure type, ion dissolution could be an important determinant too. Our data showed that ion release was greatest for doped, but the levels did not reach statistical significance (Figure 1).

It has been reported that Si is not an essential element for plant growth (Epstein, 1999). However, the presence of Si in the coated NP makes it important to quantify the Si uptake in different plant tissues. The soil type was sandy-loam with ~84% sand. The loading of the coating agent KH550 is only 1 wt% of NP. The Si content in the coated NP is negligibly small compared to that of soil. A similar scenario exists for Al content in soil (>6000 mg/kg soil), which was much higher than that in alumina doped NPs (2 wt% of NP). Consequently, due to very high background values, it is difficult to identify the effects of coating and doping on Si and Al uptake, respectively. There was a numerical decrease in Al and Si (except 1000 mg/kg doped) content in roots. Similar results were reported by Wang et al. (2013) where higher concentration of Zn (500 mg Zn/kg soil), lowered the bioavailability of Al “due to formation of ZnAl-layered double hydroxide (ZnAl-LDH).” Another, reason could be the coexistence of Si and Al with ZnO NPs, followed by adsorption onto the clay minerals (Zhao et al., 2013a). Silicon induced apoplastic binding of Al could also explain the lower translocation of Al through the shoot system of the plant (Wang et al., 2004). Moreover, alumina hydrolysis occurs in the acidic media (Balint et al., 2001) and the pH of the test media was in the basic range. That could be another reason for little or no dissolution of alumina in the soil. Nonetheless, synchrotron studies are essential to establish the relationship between NP composition and bioavailability. Ongoing speciation studies are focused on identifying the modes of interaction among bare, coated, and doped ZnO NPs with soil particles and higher plants.

From the above results, it is clear that the phyto-toxicological response of green pea from exposure to these particles was very different. At the highest concentration, bare and doped NPs showed the greatest bioaccumulation in all the parts of the plant. However, no observable sign of toxicity was observed. Therefore, it is evident that the amount of zinc present in compound/particles is not the only determining factor for NP toxicity; the form (bare, coated, and doped) of ZnO NPs also plays a crucial role.

### Chlorophyll and carotenoids in leaf

**Figure 5:**
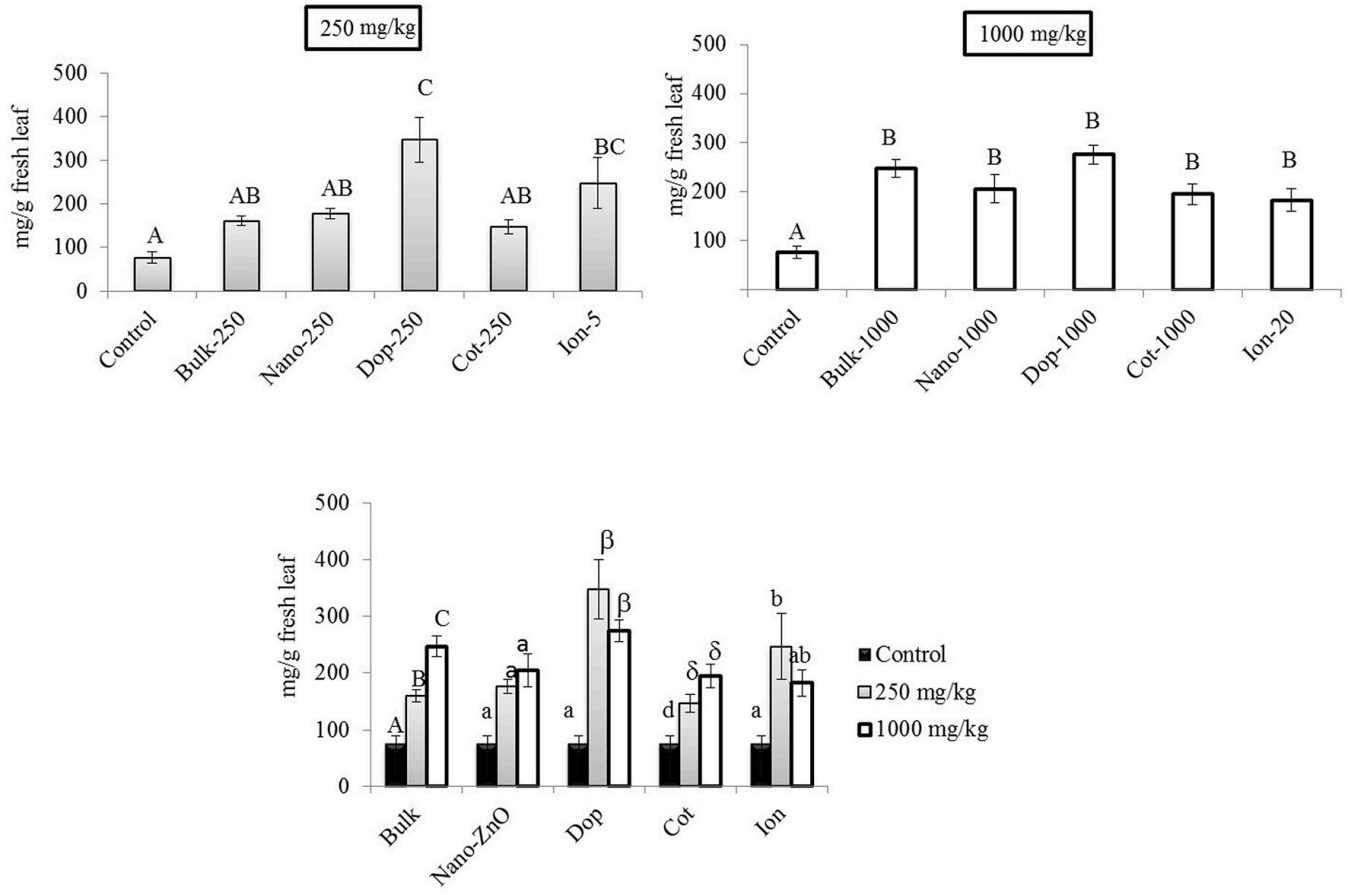
**Chlorophyll-*a* concentrations in leaf tissues**. Top graphs show the effects of different NP treatments at 250 and 1000 mg/kg exposure, respectively. The bottom graph shows the comparison among control, 250, and 1000 mg/kg treatments for each type of treatment separately. Bars are mean ± SE. Bars with same letters/symbols represent no statistical significance at *p* ≤ 0.05. Upper case, lower case, and symbols are mutually exclusive.

At 250 mg/kg, the amount of Chl-*a* increased with Zn exposure, although statistically significant increases were observed only with doped and ion treatments (3.2x–4.5x), compared to control (Figure 5). At 1000 mg/kg, all treatments resulted in 2.4–3.6 fold significant increases in Chl- *a*, compared to control, although there were no significant differences among the types of Zn amendments (Figure 5). Interestingly, there were no differences in the amount of chlorophyll-*b* (Chl-*b*) with Zn exposure (Figure S4). Similar to the leaves at 250 mg/kg, the total carotenoid content trended upward with Zn exposure but only the doped and ion treatment enhancements (10x and 7x, respectively) were of statistical significance (Supporting Information Figure S5). The same trend was evident at 1000 mg/kg but only the bulk and doped particles resulted in statistically significant increases.

Our findings are in good agreement with previous reports. For example, Prasad et al. (2012) reported higher chlorophyll content in peanut at 1000 mg/kg ZnO NP (25 nm) treatment. No effect on Chl-*b* in corn was observed at 400 mg/kg ZnO (Zhao et al., 2013a). Zhao et al. reported an increasing trend (but statistically insignificant) in total chlorophyll content in cucumber (*Cucumis sativus*) treated with 400 and 800 mg/kg bare-ZnO NP in soil (Zhao et al., 2013b). Zinc is an essential micronutrient in plants (Hansch and Mendel, 2009) but above a “threshold” concentration, the element can generate toxicity in different plant species (Broadley et al., 2007; Zhao et al., 2013b). For instance, Kupper et al. (1996) reported that zinc can substitute the central metal atom magnesium (Mg^2+^) in chlorophyll, causing a breakdown of the photosynthetic process. It has been reported that above 200 mg/kg (threshold value) in leaf tissues, *Bacopa monniera* and *Lolium perenne* L. *cv* Apollo showed phytotoxicological responses (Ali et al., 2000; Bonnet, 2000). In our study, the maximum Zn concentration in leaf was < 300 mg/kg DW. This value is likely less than the threshold Zn tolerance value (not determined here) for green pea leaves under our particular growth condition. Moreover, at 1000 mg/kg, carotenoid concentrations increased up to 9 fold, compared to control. Carotenoids are photo-absorbing pigments which might have protected Chl-*a* from photooxidation (Lichtenthaler, 1987). In leaf tissues, the unchanged (Chl-*b*) or increased (Chl-*a*, carotenoids) pigment content clearly suggests little or no toxicity to photosynthetic pigment production with Zn exposure. However, these findings may not exclude the possibility of damage to other components of the photosynthetic apparatus, e.g., electron transport chains and photosynthetic enzyme activities. Further biochemical investigations are warranted to evaluate the effects of ZnO NP exposure on other complex photosynthetic components.

### Effects of NPs on green pea seed quality

**Figure 6:**
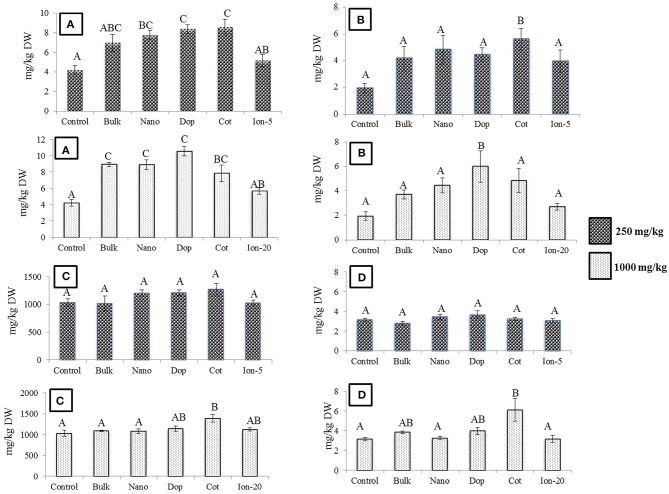
**(A)** Zinc **(B)** Iron **(C)** Phosphorus **(D)** Manganese bioaccumulation in seeds at 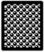 250 and 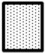 1000 mg/kg treatments. Bars are mean ± SE. Bars with same letters represent no statistical significance at *p* ≤ 0.05.

Exposure to Zn, regardless of type, generally had little effect on the green pea pod characteristics. The pod length, pod weight, and number of seeds per pod did not change as a function of treatment, with the exception of doped 250 mg/kg nanoparticles (data not shown). Here, the number of seeds per pod decreased by 33% compared to that of bare ZnO NP treatment. Unlike bulk treatments, bare, doped, and coated NPs showed increase in Zn uptake at 250 mg/kg treatment, compared to control (Figure 6). At 1000 mg/kg, the Zn content increased by 2–2.5 times in all NP and bulk treatments as compared to control. The ionic treatments did not show any significant change in Zn uptake at 5 mg/kg or 20 mg/kg. Concentrations of Cu, Mg, and K in the seed did not change significantly with Zn exposure (data not shown). The Fe level was significantly elevated by the coated (250 mg/kg) and doped (1000 mg/kg) treatments. In addition, at 1000 mg/kg coated treatment, P and Mn were significantly increased (Figures 6B–D).

**Figure 7:**
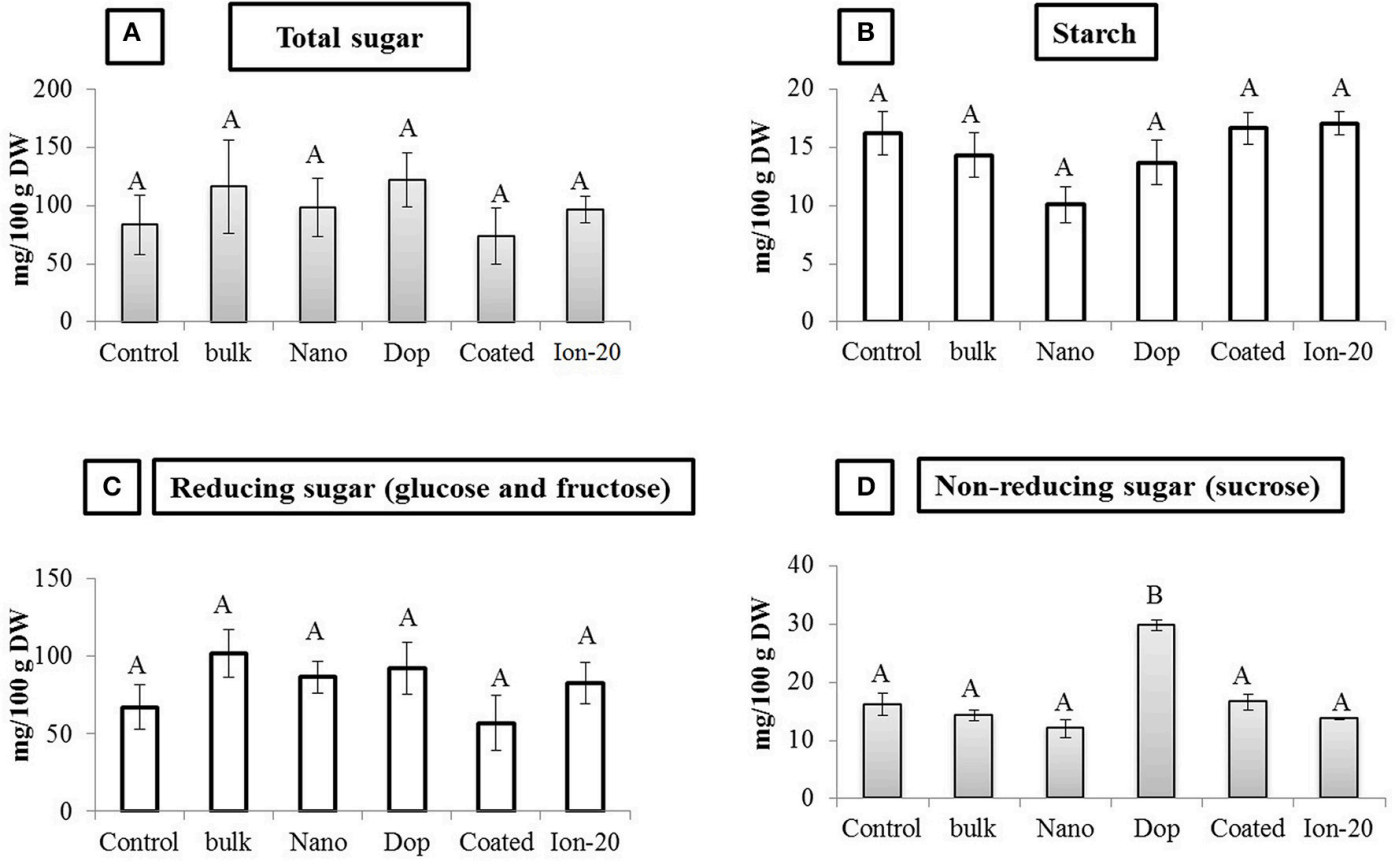
**Carbohydrate profile in seed**. **(A)** Total sugar, **(B)** Starch, **(C)** Reducing sugar, and **(D)** Non-reducing sugar contents in seed. Bars are mean ± SE. Bars with same letters represent no statistical significance at *p* ≤ 0.05.

Overall, Zn exposure, regardless of type or concentration, had little impact on the protein or carbohydrate profile of the green pea seeds. The amount of acid-soluble (glutelin), salt-soluble (globulin), water-soluble (albumin), and alcohol-soluble (prolamin) protein fractions remained unaltered in all treatments (Figure S6). There was a decrease in glutelin amount (50%) at 1000 mg/kg doped treatment, compared to control, but due to large variability and modest replicate numbers, the decrease was statistically insignificant. The amount of total sugar, starch, reducing sugars (glucose and fructose), and non-reducing sugar (sucrose) also remained largely unaltered. The exception was the 1000 mg/kg doped NP treatment where the sucrose content of pea seeds was significantly increased by 1.8 fold compared to all other treatments (Figure 7). Higher sucrose concentration in green pea at 1000 mg/kg doped treatment may be less of concern for seed quality but more problematic as an indicator of plant stress (Koch, 2004; Levitz, 2004; Zhao et al., 2014b). It has been reported that reducing and non-reducing sugars can contribute to the signaling pathways related to stress (Koch, 2004; Levitz, 2004; Zhao et al., 2014b).

As mentioned earlier, green pea plants were chosen to evaluate the effects of NP exposure because of the crop worldwide production and consumption. Green pea seeds are rich in protein, certain minerals, and vitamins and have modest calorific content (Iqbal et al., 2006). Raw green peas are excellent source of vitamin K, C, B1, B9, A, B6, B3, and B2. The crop is also rich in Mn, P, Mg, Cu, Fe, Zn, and K (Iqbal et al., 2006). Among major legumes (i.e., lentil, green peas, and common bean, among others), green pea is the second best protein source (24.9/100 g raw green pea, Iqbal et al., 2006). It has been reported that a cup of raw green peas (= 137.75 g) provides 30.3% fiber, 14.7% of protein, and only 6% calories as measured against typical daily nutritional values (Iqbal et al., 2006). There are very few reports available in the literature investigating the effect of nanoparticle exposure in soil under field-like conditions on pea seed quality. Several similar studies have been published focusing on bare-ZnO and CeO_2_ NPs exposure. For instance, Rico et al. (2014) treated wheat plants at 0, 125, 250, and 500 mg/kg soil, and found changes in nutrient content (S and Mn), amino acid, and fatty acid profiles upon exposure to CeO_2_. Our findings agree well with Priester et al. (2012) where a 2.5 fold increase in zinc uptake by soybean pods was observed upon exposure to 500 mg/kg bare-ZnO NP as compared to controls. Peralta-Videa et al. (2014) found increased zinc concentration in soybean pods at 50, 100, and 500 mg/kg bare-ZnO treatments. Moreover, at “medium” concentration (100 mg/kg), significant bioaccumulation of Cu and Mn in soybean pods were also observed. Similarly, Zhao et al. (2014b) reported that treatment with 400 and 800 mg ZnO NP/kg soil resulted in changes of micronutrient and carbohydrate content without any alteration in protein profile of cucumber fruit. Elevated levels of Zn in the seeds was likely due to the enhanced mobility of Zn^2+^ ions (Broadley et al., 2007; Wang et al., 2013) generated from the dissolution of NPs in soil. In terms of cellular uptake, there are different transporter genes and pathways present, which regulate the mobility of different metals across the plasma membrane. For example, Mn transport is regulated by natural resistance-associated macrophage protein (*Nramp*) transporters and zinc-regulated transporter/iron-regulated transporter (*ZRT/IRT1*)-related protein (*ZIP*) transporters, among others (Pittman, 2005). Currently, we have no information regarding the interaction among specific metal transporters and different NPs. As such, characterizing potential correlations between macro/macro nutrient uptake in seed with different NP exposure is too speculative with the current knowledge base. However, considering all the above data, it can be said that the mineral/nutrient concentration in the edible tissue was affected differentially by nanoparticle type, with the coated and doped ZnO exerting the greatest effects. Similarly, under high dose exposure (1000 mg/kg), doped NP altered (1.8 times higher) the carbohydrate profile (sucrose) of the seed. The implications of these NP-induced changes in fruit content/quality are currently unknown but are the subject of intense investigation.

In summary, our study investigated the comparative phytotoxicity of bare-ZnO NPs, Al_2_O_2_@ZnO, and ZnO@KH550 NPs on green pea plants in terms of biomass, element bio-accumulation, changes in leaf photosynthetic pigment, along with the changes in seed quality. Our results confirmed that, in spite of possessing larger size in the commercial form, alumina doped ZnO NPs (15 nm) have greater effects on plant and seed quality, compared to bare-ZnO NPs (10 nm). The seed quality was affected most by the doped NPs at 1000 mg/kg where nutrient content and carbohydrate profile (sucrose) changed. It was suggested in the literature that doping (Fe doped ZnO NPs) could decrease the phytotoxicological effects of bare-ZnO NPs to higher plants (Mukherjee et al., 2014b). Nevertheless, our findings clearly demonstrate that Al_2_O_3_@ZnO NP treatments exerted more negative effects on green pea when compared to bare and coated ZnO NP. Therefore, the doping agents certainly play a crucial role in the phytotoxicological responses of NP exposure to the higher plants. Although, the mechanism is unknown, ion release and coating facilitated uptake of intact NPs are possible pathways of concern. Additional study into the broader implications of NP doping and coating type on food safety and on the fate and disposition of these materials in the environment is warranted.

## Supporting information

Two tables listing details on particle characterization and elemental composition of native and 1:1 soil. Six figures describe different physiological and biochemical parameters of root, stem, leaf, and seeds.

### Conflict of interest statement

The authors declare that the research was conducted in the absence of any commercial or financial relationships that could be construed as a potential conflict of interest. The handling editor Nelson Marmiroli declares that, despite hosting the Research Topic “Nanotoxicology and environmental risk assessment of engineered nanomaterials (ENMs) in plants” together with co-author Jason C. White, the review process was handled objectively.
