# Supplementary material for: Differential Toxicity of Bare and Hybrid ZnO Nanoparticles in Green Pea (Pisum sativum L.): A Life Cycle Study
Source: Front Plant Sci. 2016 Jan 12;6:1242. doi: 10.3389/fpls.2015.01242 (PMC4710101; doi:10.3389/fpls.2015.01242)
Supplement: Supplementary file 1 [file DataSheet1.docx]

**Differential toxicity of bare and hybrid ZnO nanoparticles in green pea (*Pisum sativum* L.): A life cycle study**

Arnab Mukherjee^1,3,5^, Youping Sun^4^, Erving Morelius^1,3^, Carlos Tamez^1,3^, Susmita Bandyopadhyay^1,3^, Genhua Niu^4^, Jason C. White^5^, Jose R. Peralta-Videa^1,2,3,^, and Jorge L. Gardea-Torresdey^1,2,3^*

^1^Environmental Science and Engineering, The University of Texas at El Paso, 500 West University Ave., El Paso, TX 79968, USA. E-mail: jgardea@utep.edu; Fax: +1 915-747-5748; Tel: +1 915-737-5359

^2^Department of Chemistry, The University of Texas at El Paso, 500 West University Ave., El Paso, TX 79968, USA

^3^University of California Center for Environmental Implications of Nanotechnology (UC CEIN), The University of Texas at El Paso, 500 West University Ave., El Paso, TX 79968, USA

^4^Texas A&M AgriLife Research Center at El Paso, 1380 A&M Circle, El Paso, TX 79927, USA

^5^Department of Analytical Chemistry, The Connecticut Agricultural Experiment Station, 123 Huntington Street, New Haven, Connecticut 06504, United States

* **Correspondence**. Prof. Jorge Gardea-Torresdey, Department of Chemistry, The University of Texas at El Paso, 500 West University Ave., El Paso, TX 79968, USA. Tel: 915 747-5359; fax: 915 747 5748. E-mail address: jgardea@utep.edu

Journal: Frontiers

Prepared: 12/17/2015

9 pages in length, including 2 tables and 6 figures.

**Table S1.** Zeta potential, pH, hydrodynamic diameter of different particles. Each measurement has three replicates. Data are mean±SE (*p* ≤ 0.05). Same letters or symbols represent no statistically significant difference within column.

**Zeta potential (mV)**

| Concentration  (mg/L) | Bare-ZnO | KH550 coated | Alumina-doped | Bulk |
| --- | --- | --- | --- | --- |
| 250 | 23.6±3.89^a^ | -15.3±7.57^b^ | 27.7±4.89^a^ | 28.7±4.55^a^ |
| 1000 | 20±4.57^a^ | -26.4±7.09^b^ | 23.9±3.74^a^ | 27.7±5.36^a^ |

**pH**

| Concentration  (mg/L) | Bare-ZnO | KH550 coated | Alumina-doped | Bulk |
| --- | --- | --- | --- | --- |
| 250 | 8.0±0.01^α^ | 7.9±0.03^α^ | 7.7±0.05^α^ | 7.9±0.02^α^ |
| 1000 | 8.5±0.01^α^ | 8.0±0.02^α^ | 7.7±0.09^α^ | 7.9±0.08^α^ |

**Size (nm)**

| Concentration  (mg/L) | Bare-ZnO | KH550 coated | Alumina-doped | Bulk |
| --- | --- | --- | --- | --- |
| 250 | 397.5±25.3^A^ | 526.6±14.2^C^ | 362.2±20.7^A^ | 1627±198.9^D^ |
| 1000 | 290.9±20.2^B^ | 608.5±11.9^C^ | 244.1±25.6^B^ | 9324±236.8^E^ |

**Table S2.** Elemental analysis of native and 1:1 native soil: potting mix. Samples were analyzed in three replicates. Data are mean±SE. Across rows, values with an asterisk (*) are significantly different (*p* ≤ 0.05).

| Parameters | Native soil  (mg/kg) | 1:1 soil  (mg/kg) |
| --- | --- | --- |
| Zn | 39.4±2.4 | 87.2±4.8* |
| Al | 7542±43 | 6124±178 |
| K | 8713±249 | 12460±89* |
| Ca | 10784±100.9 | 12716±247 |
| Fe | 7515±48* | 4578±167 |
| Mg | 2711±145 | 4591±57* |
| S | 719±47 | 1451±41* |
| Mn | 57±9.14 | 89.14±4.9* |
| P | 39±1.3 | 108±4.8* |
| Cu | 14.5±4.1 | 19.0±0.9 |
| Mo | 1.1±.09 | 2.9±0.2* |


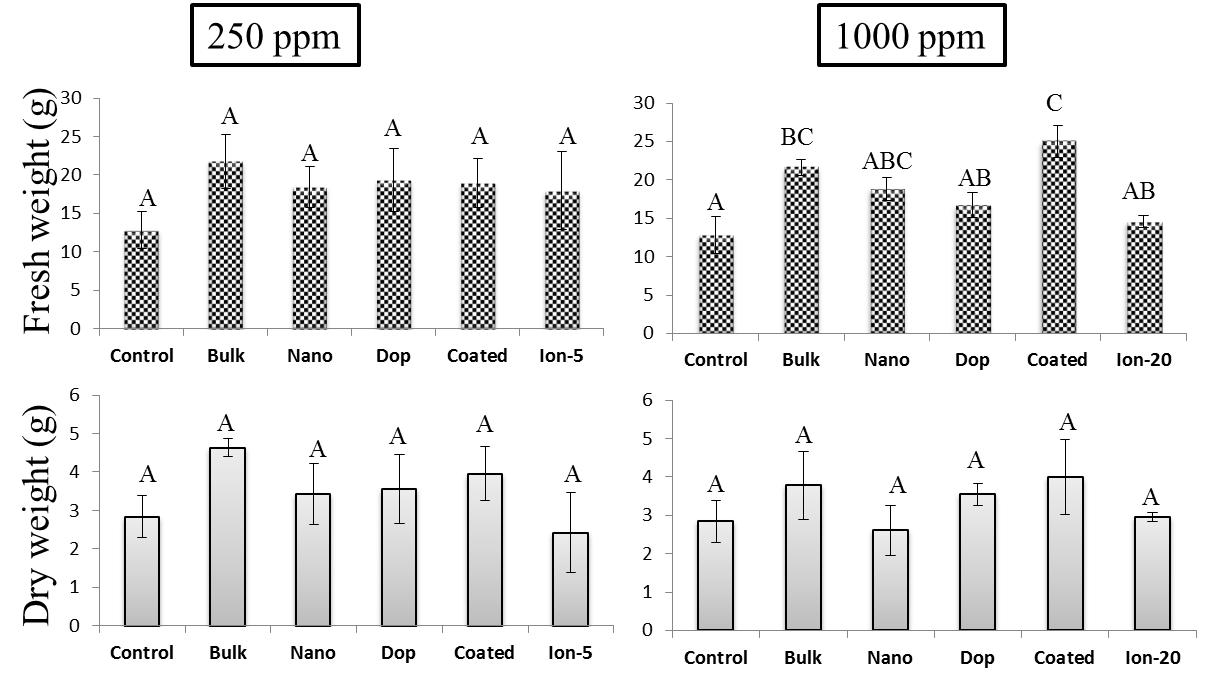


**Figure S1.** Fresh and dry mass of the total plants. Here ppm= mg ZnO NP per kg soil. Bars are mean ± SE. Bars with same letters are not significantly different *p* ≤ 0.05.


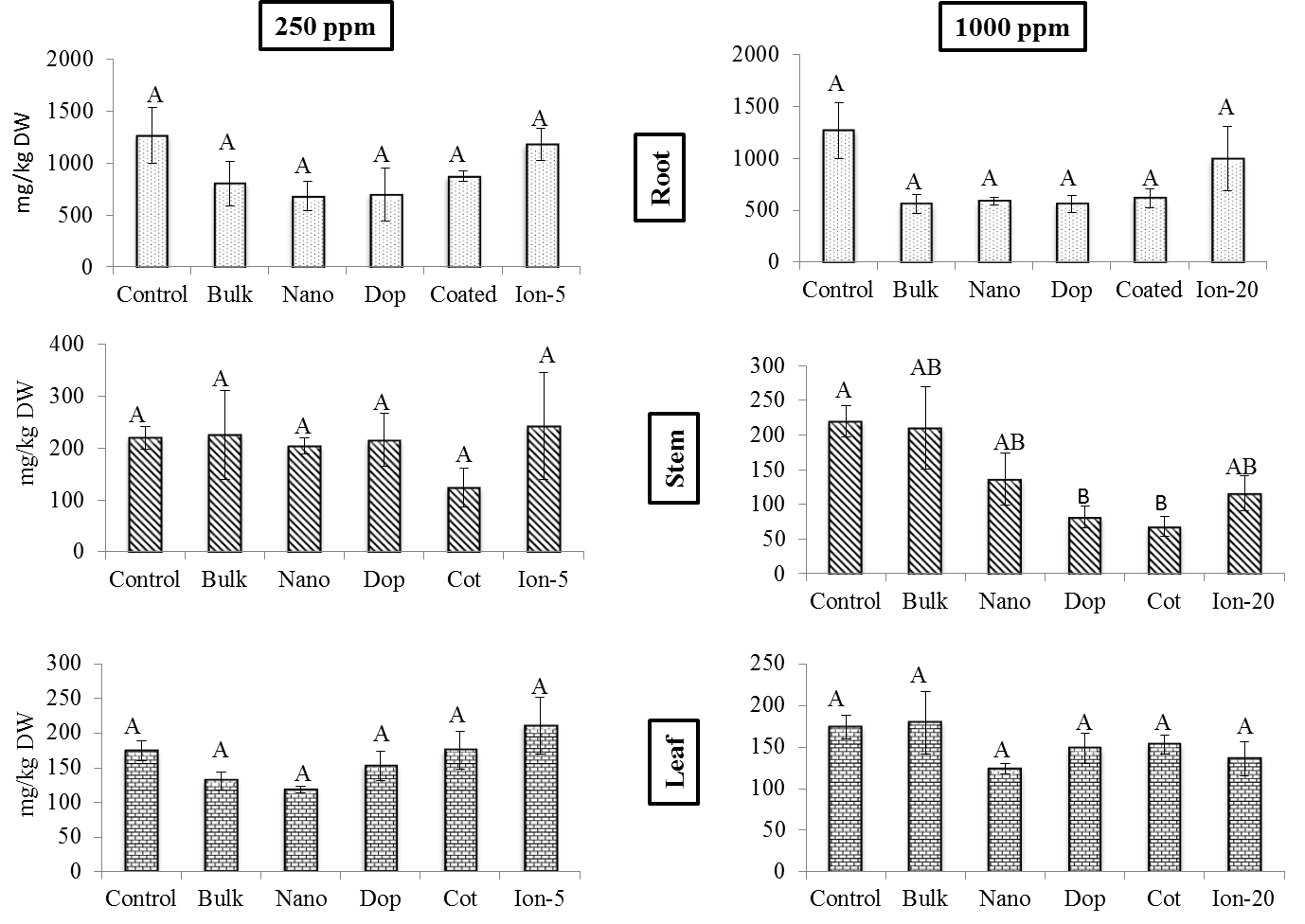


**Figure S2.** Aluminum bioaccumulation in root, stem, and leaf tissues. Here ppm= mg ZnO NP per kg soil. Bars are mean ± SE. Bars with same letters represent no statistical significance at *p* ≤ 0.05.


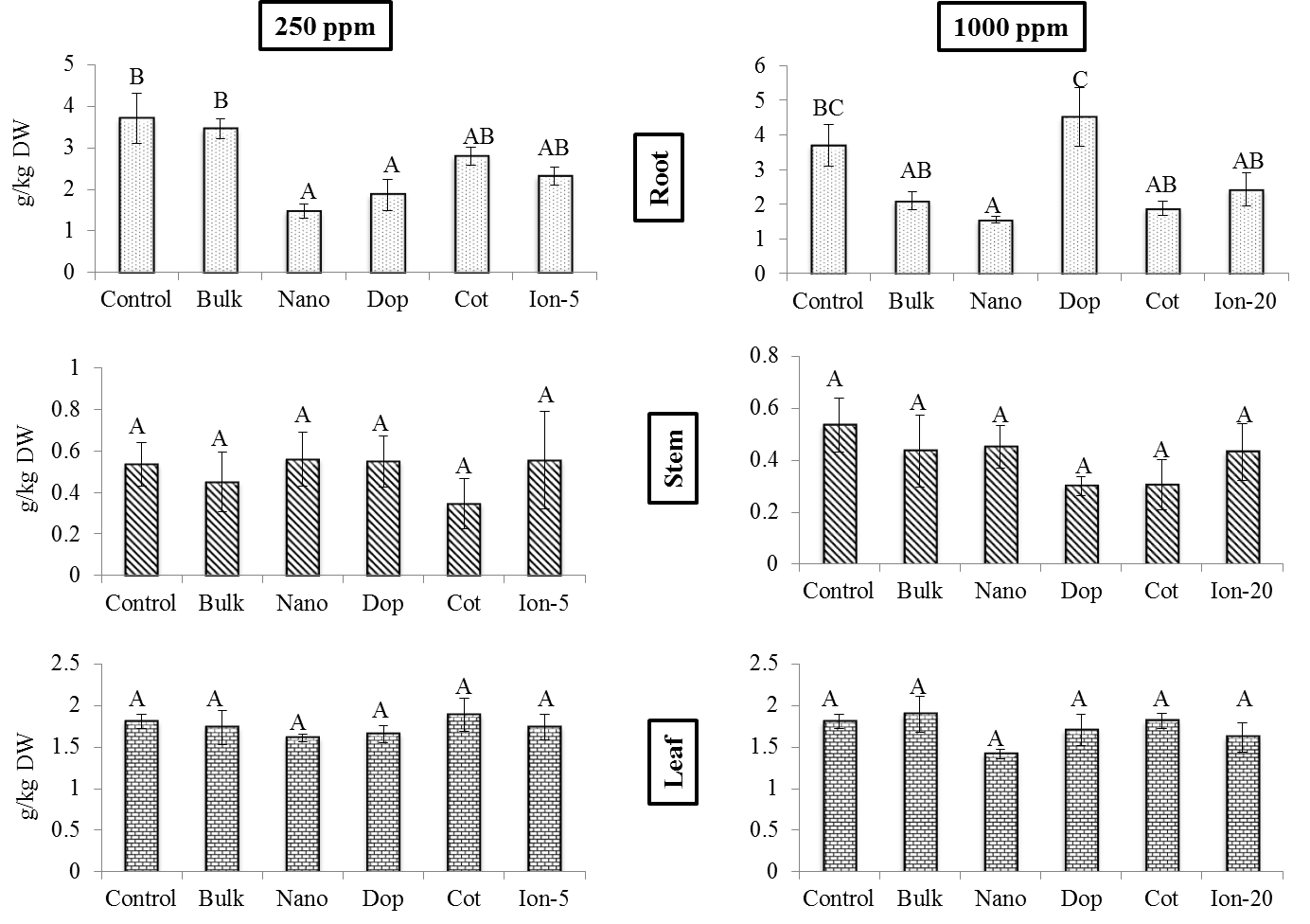


**Figure S3.** Silicon bioaccumulation in root, stem, and leaf tissues. Here ppm= mg ZnO NP per kg soil. Bars are mean ± SE. Bars with same letters represent no statistical significance at *p* ≤ 0.05. Upper case, lower case, and symbols are mutually exclusive.


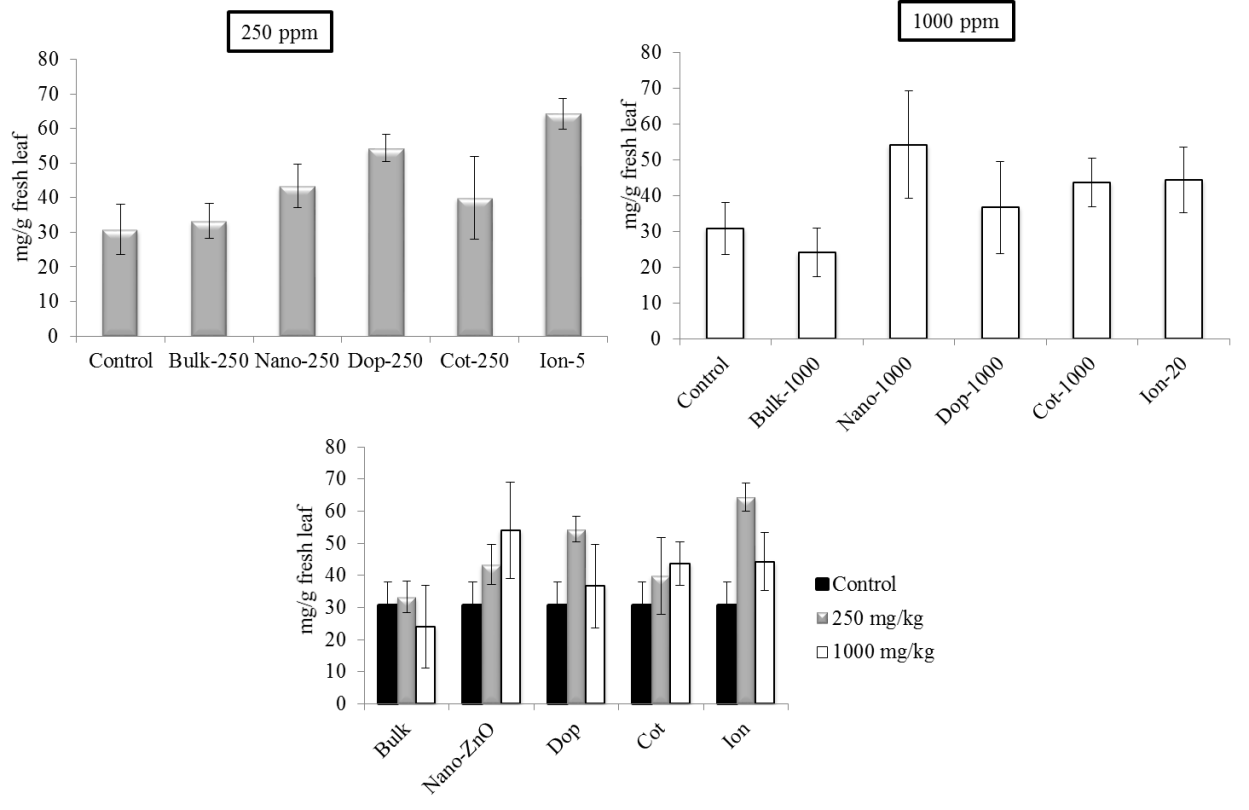


**Figure S4.** Chlorophyll-*b* concentrations in leaf tissues. Top graphs show the effects of different NP treatments at 250 and 1000 mg/kg exposure, respectively. The bottom graph shows the comparison among control, 250, and 1000 mg/kg treatments for each type of treatment separately. Here ppm= mg ZnO NP per kg soil. Bars are mean ± SE. Bars with no letters/symbols represent no statistical significance at *p* ≤ 0.05.


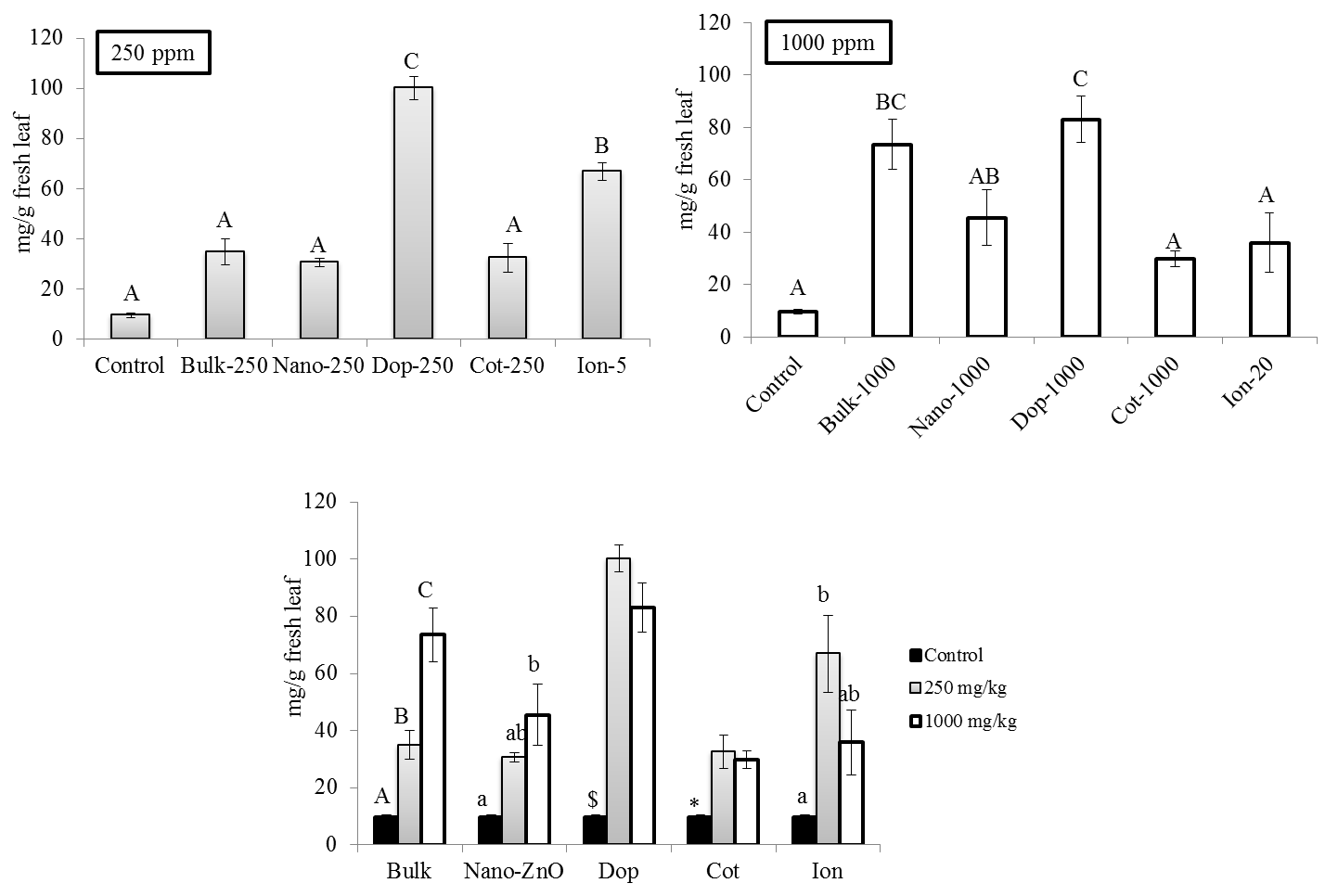


**Figure S5.** Total carotenoid concentrations in leaf tissues. Top graphs show the effects of different NP treatments at 250 and 1000 mg/kg exposure, respectively. The bottom graph shows the comparison among control, 250, and 1000 mg/kg treatments for each type of treatment separately. Here ppm= mg ZnO NP per kg soil. Bars are mean ± SE. Bars with same or no letters/symbols represent no statistical significance at *p* ≤ 0.05.


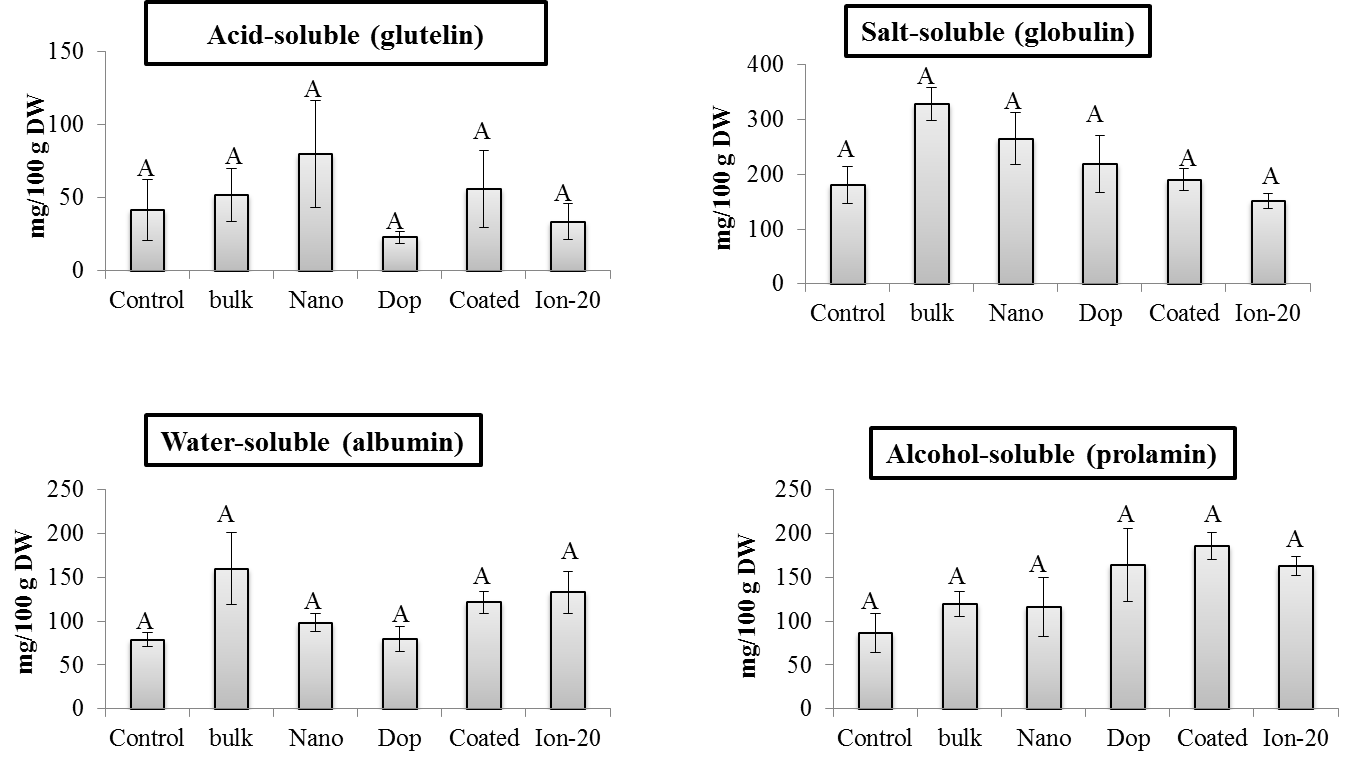


**Figure S6.** Amount of different protein fractions in seed. Bars are mean ± SE. Bars with same letters represent no statistical significance at *p* ≤ 0.05.
